# Fighting COVID-19 at the Expense of Malaria in Africa: The Consequences and Policy Options

**DOI:** 10.4269/ajtmh.20-1181

**Published:** 2020-11-11

**Authors:** Abdullahi Tunde Aborode, Kenneth Bitrus David, Olivier Uwishema, Agbendeh Lubem Nathaniel, Jegede Oluwatoyin Imisioluwa, Sherifdeen Bamidele Onigbinde, Fozia Farooq

**Affiliations:** 1 Healthy Africans Platform, Research and Development Hub, Ibadan, Nigeria;; 2 Brain Builders Youth Development Initiative, Research Directorate, Ilorin, Nigeria;; 3 Hull York Medical School, University of Hull, Hull, United Kingdom;; 4 Faculty of Pharmaceutical Sciences, Kaduna State University, Kaduna, Nigeria;; 5 Department of General Medicine, Karadeniz Technical University, Trabzon, Turkey;; 6 Oli Health Magazine Organization (OHMO), Kigali, Rwanda;; 7 BRAC James P. Grant School of Public Health, BRAC University, Dhaka, Bangladesh;; 8 Department of Human Kinetics and Health Education, Adekunle Ajasin University, Akungba Akoko, Nigeria;; 9 Department of Biochemistry, Federal University of Agriculture, Abeokuta, Nigeria;; 10 Faculty of Pharmaceutical Science, University of Sindh, Jamshoro, Pakistan

## Abstract

Malaria remains a major global health burden, killing hundreds of thousands annually, especially in sub-Saharan Africa. In December 2019, a novel illness termed COVID-19, caused by SARS-CoV-2, was reported in China. This disease soon spread around the world and was declared a pandemic by the WHO on March 11, 2020. Considering that the malaria burden is high in many low-income tropical countries with little capacity to fund malaria control and eradication programs, the fight against malaria in these regions is likely to be hindered by COVID-19. Indeed, access to health care has generally been limited during the pandemic, whereas malaria interventions, such as seasonal malaria chemoprevention, and distribution of long-lasting insecticide-treated bed nets, have been suspended because of lockdowns. Likewise, the repurposing of antimalarials for the treatment of COVID-19 and a shift in focus from the production of malaria rapid diagnostic tests to COVID-19 rapid diagnostic tests are causes for concern in malaria-endemic regions. COVID-19 has disproportionately affected developed countries, threatening their capacity to aid in malaria control efforts. Here, we address impacts of the COVID-19 pandemic on the management and control of malaria in Africa.

## INTRODUCTION

Malaria remains a major health burden globally. In 2018, an estimated 228 million malaria cases and 405,000 deaths occurred worldwide, with sub-Saharan Africa bearing the greatest brunt.^1^ Unfortunately, in December 2019, a novel illness termed COVID-19, caused by SARS-CoV-2,^2^ was reported in Wuhan, China.^3^

COVID-19 rapidly spread into a global pandemic, as declared by the WHO on March 11, 2020. As of October 23, 2020, there were more than 26 million confirmed cases of COVID-19 and more than six million active cases in 215 countries and territories; and fatalities exceeded eight hundred thousand.^4^

Considering that the malaria burden is highest in low-income countries with little capacity to fund control and elimination programs,^5^ the fight against malaria is likely to be impacted negatively by the COVID-19 pandemic. COVID-19 at the time of writing this perspective has been disproportionately affecting the developed countries.^6,7^ Consequently, these countries that mostly fund malaria control and interventions may devote more disease control resources inward, and this could jeopardize resources for malaria control efforts in the low-income regions.^8–10^

Here, we present our views on the potential collateral impact of COVID-19 on malaria and the likely implications of the COVID-19 pandemic on malaria, especially in Africa.

## COVID-19 AND MALARIA IN AFRICA

The COVID-19 pandemic has substantially impacted the control of infectious diseases, thereby impeding recognized program addressing malaria and other infectious diseases.^11^ The gains of Africa in decreasing disease and death have been significantly attributed to expanding the distribution of insecticide-treated bed nets,^12^ indoor spraying of residual insecticides, access to early diagnosis, and more effective antimalarial treatments,^11^ coupled with targeted interventions such as intermittent preventive treatment in pregnancy and seasonal malaria chemoprevention. This combined approach has been enabled by financial and global commitment to malaria control and elimination, encouraged by future targets, such as reducing malaria globally by > 90% by 2030 (compared with 2015), eliminating malaria from the Asia Pacific by 2030 and Africa being largely malaria free by 2050.

Recently, progress in reducing the global burden of malaria has stalled. In 2018, there were an estimated 228 million cases, compared with 214 million in 2015, and more than 400,000 deaths.^13^ The emergence of drug-resistant parasites and insecticide-resistant mosquitoes, lack of universal access to malaria prevention and treatment, and the lack of a highly effective vaccine constitute the challenges in eradicating Malaria. Malaria funding is below what is required to achieve global goals, and many countries face competing health priorities in the context of severely constrained resources.^14^ In this regard, the emergence and spread of COVID-19 may hinder malaria control and consequently reverse successes in many malaria-endemic countries.

With lockdowns in many countries, supply chains of health commodities were cut short. This could lead to shortages of medicines across the world, including treatments for critical illness. This is of greatest concern for low- and middle-income countries that still bear the brunt of the burden of communicable diseases such as malaria, HIV/AIDS, and tuberculosis. The impact of COVID-19 on pharmaceutical supply chains could imperil progress made against malaria in malaria-endemic countries.

In the past, restricting malaria control activities has been accompanied by resurgence in malaria morbidity and mortality.^15^ This has occurred when interventions were reduced because of funding constraints or disrupted by war, disaster, or conflict.^16^ Following the termination of a dichlorodiphenyltrichloroethane program in Indonesia in the 1960s, annual malaria cases rose from < 6,000 to 346,000 per year. Countries that are drawing toward elimination of malaria face similar risks of resurgence if current programs are significantly disrupted by the COVID-19 pandemic.

Disruption of economic activities and health system failure are responsible for rise in morbidity and mortality from infectious diseases. The COVID-19 pandemic has crumbled socioeconomic activities which could result in the malaria resurgence. Deaths from malaria, HIV infection, and tuberculosis in West Africa increased because of Ebola epidemic in 2017.^17^ This could be attributed to deaths of healthcare workers, overwhelmed health facilities, and fear of contracting disease at health facilities.^17,18^

These same factors are evident during the COVID-19 pandemic. The Ebola epidemic disrupted the distribution of insecticide-treated bednets (ITNs), potentially resulting in increased malaria transmission, whereas poor access to malaria treatment was associated with dramatic increases in deaths in children.^19^ Management of COVID-19 must be combined with support for malaria treatment and prevention programs.^20^ This will need to be maintained until COVID-19 is arrested. Established malaria intervention programs and innovative approaches such as targeted mass development administration programs and enhanced distribution of ITNs will play a key role in preventing dramatic increases in malaria deaths,^21^ but the disruption of economic activities due to COVID-19 challenges the implementation and prioritization of these programs.^22^ The effects of the pandemic on the fight against malaria and other infectious diseases could be disastrous, threatening to reverse progress made over the years. New modeling studies by the WHO show that if health systems collapse or treatment and prevention services are interrupted, the death toll from malaria could double over the years.^19^

Although COVID-19 is less often severe in children and pregnant women^20,23^ than in old people, these groups would bear a disproportionate burden of excess malaria mortality arising from COVID-19–related disruption of health systems and malaria control programs, particularly in sub-Saharan Africa. Recently, the Malaria Atlas Project modeled these potential impacts in Africa for the WHO’s Global Malaria Program.^24^ A range of scenarios was considered, such as ceasing ITN distribution campaigns planned for 2020, reductions of routine ITN distribution, and reduced access to effective antimalarial drugs. In the worst-case scenario, a 75% decrease in ITN distribution coupled with a 75% decrease in access to artemisinin combination therapies (ACTs) was predicted to result in a 22% increase in malaria cases, and doubling of malaria deaths within a year to 769,000,^25^ 70% of them in children younger than 5 years.^26,27^These models do not include additional increases in malaria that could result from disruptions in the distribution of (which currently protects 19 million children in 12 countries)^28^ and intermittent preventive treatment in pregnancy seasonal malaria chemoprevention (which protects pregnant women and their babies in 36 African countries from malaria in pregnancy and low birth weight).^13^ With the high prevalence of malarial cases in Africa, there is need for the direct response to COVID-19 to be integrated with efforts to control malaria.^29^

Malaria healthcare workers are frequently reassigned from malaria intervention programs to support COVID-19 response efforts and are themselves at high risk of COVID-19 in Africa.^28,30^ Regardless of the risks, adequate personal protective equipment is often lacking, and workers suffer the stigma of being potential sources of viral infection.^8,31^ Malaria elimination and intervention programs must reach marginalized groups living in remote and border areas,^32^ but these programs face setbacks because of logistic or economic reasons associated with COVID-19, putting communities at risk.^33,34^ These complex issues pose a serious challenge to the provision of health care and monitoring for malaria and reduce elimination efforts.

## REPURPOSING ANTIMALARIALS FOR THE TREATMENT OF COVID-19

Artemisinin combination therapy is used to treat malaria in most endemic countries, but artemisinin resistance emerged in Southeast Asia.^35,36^ Recently, Madagascar reported a tonic of *Artemisia annua*, a plant that contains artemisinin, as a potential cure for COVID-19.^37^ The plant extract is likely to be officially or illicitly adopted for COVID-19 therapy by many countries in a desperate attempt to avert COVID-19–related deaths despite the WHO warning against its use without establishment of efficacy.

Although the WHO discourages the use of the *Artemisia* herb against malaria,^38^ alternative medicine and pharmaceutical industries are likely to exploit the plant for SARS-CoV-2 treatment. Any widespread use of *Artemisia* for SARS-CoV-2 infections may potentiate the development of artemisinin resistance, endangering the efficacies of first-line ACT regimes.^39,40^ The antimalarial drugs chloroquine and hydroxychloroquine are heavily used for the management of SARS-CoV-2 despite limited evidence for efficacy^41^ and against the advice of the WHO.^42^

The use of chloroquine to treat malaria had been officially discontinued in most endemic areas owing to drug resistance, but recent studies have shown decreasing resistance and suggested reintroduction for malaria treatment.^43,44^ The heavy use of chloroquine and its derivatives in COVID-19 treatment is likely to facilitate selection of chloroquine-resistant malaria parasites. This will hinder the possible use of chloroquine and its derivatives in the fight against malaria in the future, and thus potentially dampen the prospects of malaria elimination from Africa.

The world is fully focusing on COVID-19 at the expense of other endemic diseases, such as malaria, that also have high rates of mortality, especially in low-income countries. For instance, an outbreak of malaria in Zimbabwe resulted in at least 131 deaths during the SARS-CoV-2 lockdown in that country.^45^

Cameroon reported a substantial upsurge in malaria cases and deaths during the COVID-19 pandemic.^46^ The malaria deaths in Zimbabwe and Cameroon were attributed to shortages of malaria drugs and lack of access to medical facilities.^46^ This trend is likely to be replicated in other African countries. In view of this, the WHO is calling for attention toward malaria interventions while responding to the pandemic to avoid the unintended consequences of COVID-19 on malaria in Africa.^47^ One way to mitigate this loss of attention to malaria is to consider both COVID-19 and malaria diagnoses in cases of fever.^30,48^

## SHIFTING ANTIGEN (OR ANTIBODY)-BASED MALARIA RAPID DIAGNOSTIC TEST (MRDT) PRODUCTION TO COVID-19 RAPID DIAGNOSTIC TEST (RDT)

There is evidence that companies are shifting their mRDT production focus and repurposing the production pipelines to COVID-19 RDTs.^25^ Rapid diagnostic tests are the most widely used diagnostic tools for malaria.^49^ Therefore, the shortage of mRDTs as a direct result of these actions will potentially jeopardize the WHO test, treat, and track policy for malaria control.^11^ It is also feared a shortage of mRDTs may result in presumptive treatment of all fevers as malaria, with associated cost implications and increased risk of selection of antimalarial drug resistance.^25^

## CONCLUSION AND RECOMMENDATIONS

COVID-19 is likely to impact the global economy and health, in unmatched proportions, particularly for weak and vulnerable economies and public health systems in Africa. Increased continental disease surveillance and relevant research are required to understand the implications of the current COVID-19 pandemic on other endemic infectious diseases in Africa. The Africa Centre for Disease Control must use its leverage within the African Union to encourage the allocation of funds by member states for disease surveillance, local scientific research, and interventions. Repurposing of antimalarials and the development of diagnostics for COVID-19 must be handled in a manner that does not jeopardize the gains made in controlling malaria and other endemic diseases. Malaria diagnostics could be coupled with COVID-19 screening to avoid misdiagnosis and enable ready management of both infections. Neglecting malaria in favor of COVID-19 could prove catastrophic for global health, particularly in Africa.
